# The impact of breed and tissue compartment on the response of pig macrophages to lipopolysaccharide

**DOI:** 10.1186/1471-2164-14-581

**Published:** 2013-08-28

**Authors:** Ronan Kapetanovic, Lynsey Fairbairn, Alison Downing, Dario Beraldi, David P Sester, Tom C Freeman, Christopher K Tuggle, Alan L Archibald, David A Hume

**Affiliations:** 1 The Roslin Institute and Royal (Dick) School of Veterinary Studies, University of Edinburgh, Easter Bush, Midlothian, Edinburgh EH25 9RG, United Kingdom; 2 Institute for Medical Microbiology, Immunology and Hygiene, Technische Universität München, 81679, Munich, Germany; 3 Cancer Research UK Cambridge Research Institute, Li Ka Shing Centre, Robinson Way, Cambridge CB2 0RE, UK; 4 Innate Immunity Laboratory, School of Chemistry and Molecular Biosciences, University of Queensland, Brisbane, Queensland QLD 4072, Australia; 5 Department of Animal Science, Iowa State University, Ames, IA 50011, USA

**Keywords:** Pig, Macrophages, Microarray, Breed, Lipopolysaccharide

## Abstract

**Background:**

The draft genome of the domestic pig (Sus scrofa) has recently been published permitting refined analysis of the transcriptome. Pig breeds have been reported to differ in their resistance to infectious disease. In this study we examine whether there are corresponding differences in gene expression in innate immune cells

**Results:**

We demonstrate that macrophages can be harvested from three different compartments of the pig (lungs, blood and bone-marrow), cryopreserved and subsequently recovered and differentiated in CSF-1. We have performed surface marker analysis and gene expression profiling on macrophages from these compartments, comparing twenty-five animals from five different breeds and their response to lipopolysaccharide. The results provide a clear distinction between alveolar macrophages (AM) and monocyte-derived (MDM) and bone-marrow-derived macrophages (BMDM). In particular, the lung macrophages express the growth factor, FLT1 and its ligand, VEGFA at high levels, suggesting a distinct pathway of growth regulation. Relatively few genes showed breed-specific differential expression, notably CXCR2 and CD302 in alveolar macrophages. In contrast, there was substantial inter-individual variation between pigs within breeds, mostly affecting genes annotated as being involved in immune responses.

**Conclusions:**

Pig macrophages more closely resemble human, than mouse, in their set of macrophage-expressed and LPS-inducible genes. Future research will address whether inter-individual variation in macrophage gene expression is heritable, and might form the basis for selective breeding for disease resistance.

## Background

Macrophages are the first line of defence against many pathogens [1]. They discriminate self from non-self through the recognition of pathogen-associated molecular patterns (PAMPs) that are not present in the host. The most-studied PAMP is lipopolysaccharide (LPS), a structural component of the cell wall of gram negative bacteria recognised by toll-like receptor (TLR) 4, which elicits much of the pathology of gram-negative septicaemia. Macrophages respond to LPS with a sequential cascade of altered gene expression that leads first to inflammation and elimination of the pathogen, and then to resolution of tissue damage [2-4]. The laboratory mouse has been used extensively as a model for the study of macrophage biology and the response to pathogens. However, mice and humans differ rather fundamentally in the nature of their innate effector pathways. Even amongst strict orthologs numerous inducible genes are regulated in one species and not the other, due in large measure to differences in promoter sequences [5]. For example, human macrophages do not induce the effector enzyme, inducible nitric oxide synthase (iNOS or NOS2), which generates the toxic radical nitric oxide, but instead induce indoleamine dioxygenase (IDO) in response to LPS [6,7]. These differences are also evident when one compares gene expression profiles of mouse inflammatory models with human disease [8]. Of course, aside from divergent expression of orthologous genes, a feature of the evolution of the immune system across species, and even within species, is the gain and loss of individual genes, especially within gene families [9]. Such differences further undermine the utility of the mouse as a model.

The domestic pig (*Sus scrofa*) has been used extensively in medical research [10], and in contrast to experimental animals, is economically important; the most important meat-producing livestock species world-wide (from OECD-FAO Agricultural Outlook 2011–2020). Because of the intensive mode of production, they are highly susceptible to pathogen epidemics that can cause huge economic losses. Viral (e.g. influenza A, African swine fever, classical swine fever, porcine adenovirus, porcine respiratory and reproductive syndrome (PRRS), parainfluenza) and bacterial (e.g. *Salmonella spp*, *Yersinia enterocolitica*, *Mycoplasma hypopneumoniae, Actinobacillus pleuropneumoniae*) pathogens often target the macrophage for replication and alter their gene expression. Many of these agents are zoonotic. One advantage of mouse models is the availability of inbred lines that can be used to map disease-susceptibility loci. Pig breeds may offer some of the same advantages. Studies of viral (PRRS) and bacterial (*actinobacillus*) infections suggest that variation in disease susceptibility or pathology between breeds, or between individuals within a breed, is correlated with differences in macrophage activation [11-13]. Such breed-specific variation also offers opportunities to breed for disease resistance or tolerance.

The study of pig macrophage biology has recently been expedited by the completion of a draft genome sequence [14], comprehensive annotation of the pig immunome [9], the development of a comprehensive expression array platform [15], methodology for cultivation of macrophages [16] and identification and characterisation of subsets of monocytes [17]. Using these tools we demonstrated that pig macrophages are much more similar to human than to mouse (and correspondingly, inducible promoters are more conserved) [16], and also provided preliminary evidence for distinct gene expression profiles amongst resident tissue macrophage populations [15]. The macrophages of the lung are of particular interest because this is a major portal of pathogen entry. There is evidence that they are specifically adapted to the airway environment [15] and these cells are not readily accessed in large numbers from experimental animals.

In the current study, we have combined the available tools to extend the knowledge of the macrophage biology of the domestic pig. We have compared the expression profiles of macrophages from different tissue compartments, and their response to bacterial LPS, in multiple individuals from five divergent pig breeds. Analysis of the entire dataset using the network analysis tool Biolayout *Express*^3D^ serves to highlight clusters of genes that share regulatory patterns across genetic and cellular variation. The data identify variation between individual pigs and breeds, and confirm the similarities between pigs and humans that support the use of the pig as a more predictive model than the mouse in biomedical research.

## Results

### Preliminary characterisation of the response to LPS in different macrophage populations

**Figure 1:**
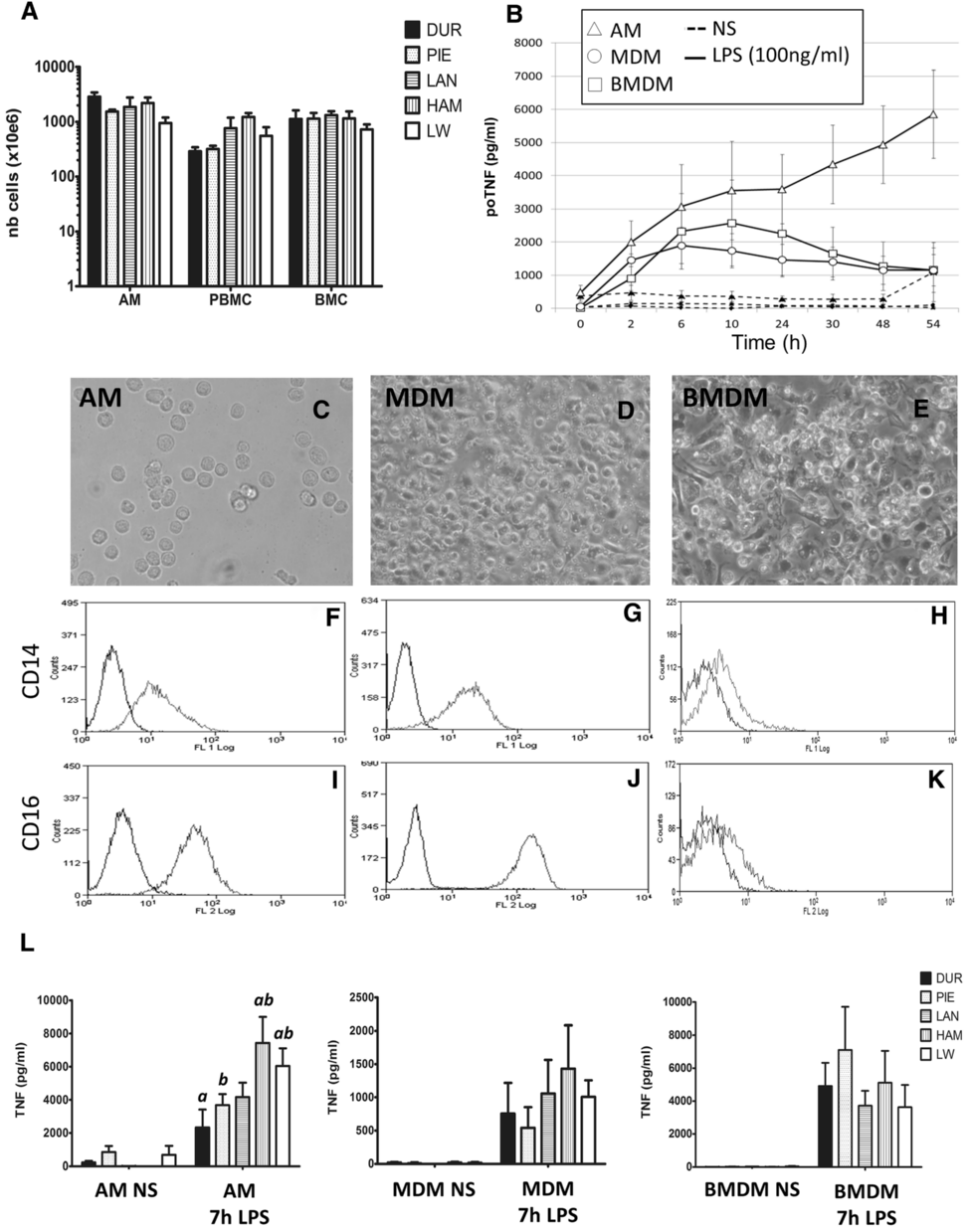
**Isolation and characterisation of macrophages from 3 different compartments. (A)** Mononuclear cells were harvested from the lungs, blood and bone-marrow. A large number of cells (alveolar macrophages, PBMC and bone-marrow cells) were harvested from the 5 breeds of pigs (Duroc, Piétrain, Landrace, Hampshire and Large White). **(B)** The 3 type of macrophages were stimulated with LPS in order to analyse the inflammatory response. Monocyte and bone-marrow cells were cultured 5–6 days with rhCSF-1 (1×10^6^ units/ml) in order to obtain macrophages. AM (Δ), BMDM (□) and MDM (○) were plated at 1×10^6^ cells/ml and left untreated (black) stimulated with 100 ng/ml of LPS (white). Supernatant was harvested at different timepoints (0, 2, 6, 10, 24, 30, 48 and 54 h) and porcine TNF was measured by ELISA (pg/ml). Morphology of AM, MDM and BMDM are pictured in **(C, D and E, respectively)** after being left overnight in complete medium with rhCSF-1. Surface expression of CD14 **(F-G-H)** and CD16 **(I-J-K)** on AM, MDM and BMDM was measured by flow cytometry. 1×10^6^ cells were stained and data was acquired from 15,000 events. The correct isotype control is shown by a dark black line and the targeting antibody by the lighter line. Data is representative of a minimum of 3 different experiments. **(L)** TNF production (pg/ml) of AM, MDM and BMDM at 0 and 7 h of LPS stimulation in function of the 5 breeds of pigs (DUR, PIE, LAN, HAM and LW). Histograms are the average of 5 individual pigs +/- SED. For AM, TNF production from DUR (a) and PIE (b) are significantly lower than HAM and LW (p<0.05).

We harvested macrophages from twenty-five pigs, five from each breed: Duroc (DU), Piétrain (PIE), Landrace (LR), Hampshire (HAM) and Large White (LW), with an average age of 9 weeks. A major advantage of the pig is that large numbers of cells can be harvested for biochemical studies; 1×10^9^ cells for each compartment (Figure 1A). To optimise the comparison, we first examined a time course of the response to LPS of alveolar macrophages (AM), bone marrow-derived macrophages (BMDM) or monocyte-derived macrophages (MDM) from Large White pigs (Figure 1B). There was a clear distinction between the CSF-1 cultured macrophages (BMDM, MDM) and AM. As noted in human and mouse macrophages, TNF production in BMDM and MDM was transient. After 10 hours, there was no further increase in supernatant TNF. By contrast, in AM, TNF production continued to rise even 54 hours post-stimulation. These preliminary studies suggested that the 7 hour time point, used in previous studies of mouse and human macrophages [5], would also provide representative coverage of the response to LPS responsive mRNAs in all of the porcine macrophage populations.

To enable the study of the macrophages from the twenty-five animals at the same time and under the same conditions, AM, PBMC and BMC were frozen as described previously [16] on the day of the harvest and used a few weeks later. PBMC and BMC were cultured for 5 to 7 days in the presence of rhCSF-1 until differentiation into macrophages [16]. The three types of macrophages were seeded at 1×10^6^ cells/ml, cultivated overnight before removing non-adherent cells, replacing the medium, and cultivating with or without LPS (100 ng/ml). Morphologically, the BMDM and MDM were more spread on the substratum by comparison to AM, where a subpopulation of cells is non-adherent (Figure 1C, D, E). Each of the populations expressed the macrophage markers CD14 and CD16, albeit at varying levels (Figure 1F-K). In order to control for the efficacy of LPS stimulation in each experiment, prior to expression profiling, TNF concentration was measured in the supernatant of the culture at 0 and 7 h (Figure 1L). With the exception of AM from HAM and LW, there were no obvious differences between the breeds in terms of the magnitude of this response. The higher production of TNF by AM from these 2 breeds appeared to be due to a higher percentage of adherent macrophages amongst the cells from the broncho-alveolar lavage, which would not interfere with the microarray analysis.

**Figure 2:**
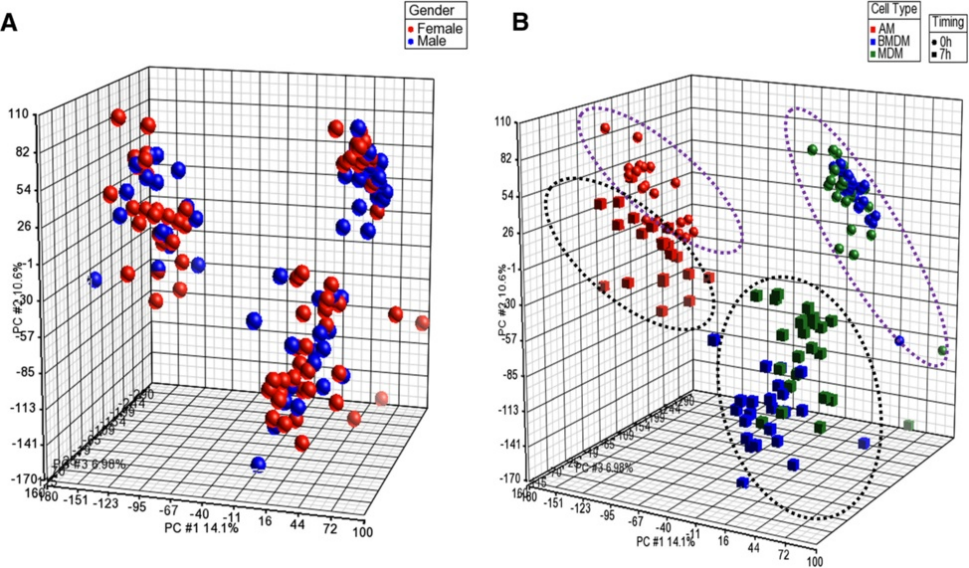
**Analysis of the effect of gender and compartment by principal component analysis (PCA).** RNA from the 25 pigs (3 types of cells, 2 time points) were hybridized with the new *Snowball* Affymetrix microarray. 140 microarrays out of the 150 were analysed using the Partek Software (10 microarrays did not pass quality check). **(A)** PCA was done on the gender of the pigs (50 males - blue, 100 females - red). No specific cluster could be observed. **(B)** PCA was also applied to the array data from the different compartments harvested (AM in red, BMDM in blue and MDM in green) and the treatment (sphere for 0 h and cube for 7 h LPS). Both compartment and time point can be clustered. Macrophages stimulated with LPS (boxes) are separated from the controls (spheres). AM (red) form a separate cluster whereas BMDM (blue) and MDM (green) are grouped together.

The mRNA was extracted from 25 pigs (from 5 breeds) from 3 different compartment (AM, BMDM and MDM) and at 2 time points and was profiled using the recently developed Snowball Affymetrix array [15]. Of 150 arrays, 10 failed the quality check. The data from the microarray are available at NCBI GEO (GSE45145). Since the pigs studied were a mixture of male and female, we investigated first whether gender has any global impact on gene expression in pig macrophages. A principal components analysis (PCA) of the data did not show any distinct clusters related to the gender of the animals (Figure 2A). Detailed analysis on the differentially regulated (DR) genes highlighted a small list of 10 genes that differed between males and females. The annotation of these probesets links them to obvious sex-dependent genes, such as USP9 Y-linked or inactive-X-specific transcript (XIST). Hence, the inflammatory response to LPS of macrophages *in vitro* is independent of the gender and we considered all the data as a single set.

### Alveolar macrophages show a distinct expression profile from BMDM and MDM

The recently published pig gene expression atlas [15] included replicates of AM, BMDM and MDM from two individual crossbred pigs, but did not compare the populations in detail. We inferred that AM were distinct from macrophages in the wall of the gut, notably in their expression of C-type lectin receptor genes.. The current dataset permits comparison in much greater depth, with the macrophages isolated from the same animals, and with 25-fold replication of the comparison. PCA analysis of the data based upon cell compartment clearly distinguishes AM from BMDM and MDM which are very similar to each other (Figure 2B).

**Figure 3:**
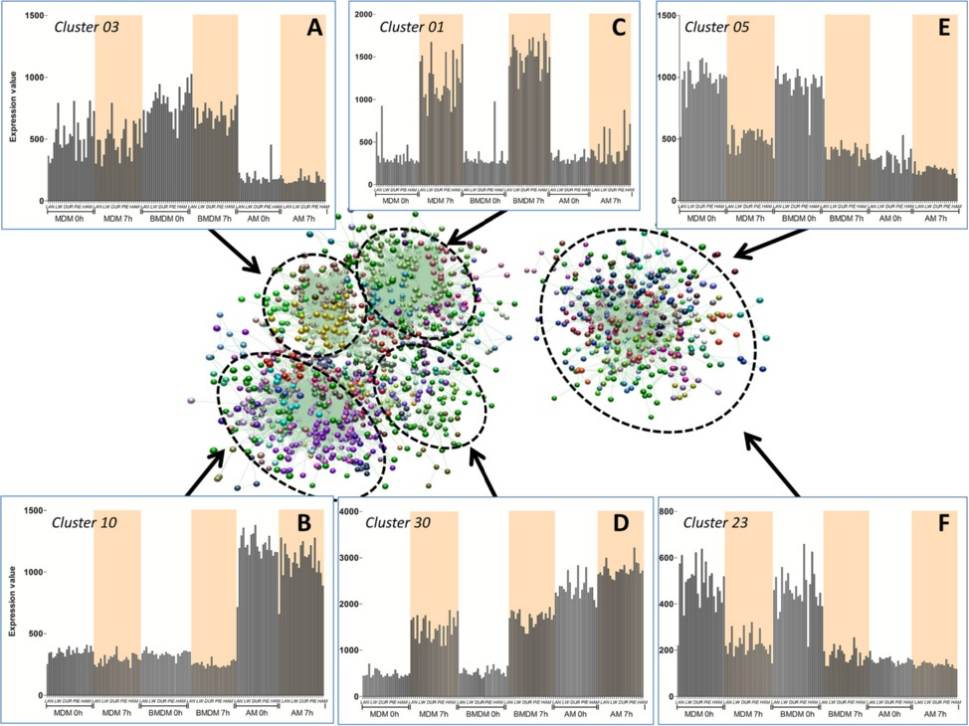
**Clusters of pig macrophage gene expression using Biolayout.** 3D visualization of a Pearson correlation (R=0.91) from the analysis of the 140 pigs micro-arrays (BMDM, MDM and AM - at 2 timepoint 0 h and 7 h). Each sphere represents an individual probeset and is composed of 5203 nodes and 29799 edges. In order to more easily differentiate the data from background noise, probesets with an expression intensity <50 in all samples have been removed from the analysis. Clustering of the graph, using the MCL algorithm (MCL = 2.2), gave a total of 505 clusters, all listed in Additional file 4. **(A-F)** To identify the different superclusters, histograms of some clusters have been included in the figure showing the average expression of genes on all condition (MDM 0 h, MDM 7 h, BMDM 0 h, BMDM 7 h, AM 0 h, AM 7 h). In these histogram plots for every condition the data for each breed are shown in the same order (LR-LW-DU-PIE-HAM). Cells treated with LPS are highlighted in orange in each graph.

To identify sets of co-expressed genes, the transcriptomic data were loaded into Biolayout Express^3D^. Using a Pearson correlation threshold cut-off of R=0.91, we obtained a graph comprising 3,586 nodes (individual probesets) made up of 505 different clusters. The clusters can be segregated broadly into two superclusters as shown in Figure 3. The larger of the two superclusters (1,470 nodes) separates into further groups with related expression profiles. One group (including cluster 03), with elevated expression in BMDM/MDM compared to AM (Figure 3A) includes genes encoding proteins involved in the cell cycle and DNA replication such as Cyclin B1, centromere proteins CENP and histones-related proteins HIST1H1A, HIST1H2AC. This most likely reflects the fact that BMDM/MDM have been stimulated to proliferate with the growth factor, CSF-1. A second group, including cluster 10, includes the genes more highly expressed in AM. As inferred previously based upon a much smaller dataset [15], it includes genes encoding the C type lectins such as MRC1, and the signalling molecules TLR4 and MAP3K2 (Figure 3B). A third group, including Cluster 1 (e.g. CCR5, CXCL10, CXCL11, DDX58, IL15), comprises genes that were up-regulated after LPS stimulation in BMDM/MDM but not at all in AM (Figure 3C). Finally, another cluster exemplified by cluster 30, (Figure 3D) which includes genes such as IL1A, CCL3L1 or TRAF3IP2, was induced in all the macrophage populations studied.

The second supercluster (407 nodes) is made up of clusters of genes down-regulated after LPS stimulation, such as cluster 05 and 23 (Figure 3E-F). These two clusters contain genes encoding proteins linked to intracellular signalling, kinase and phosphatase (PKC, PIK3IP1, TRAK2, DNM3, PLEK). The full list of clusters and the probes within them can be found in the Additional file 1.

**Figure 4:**
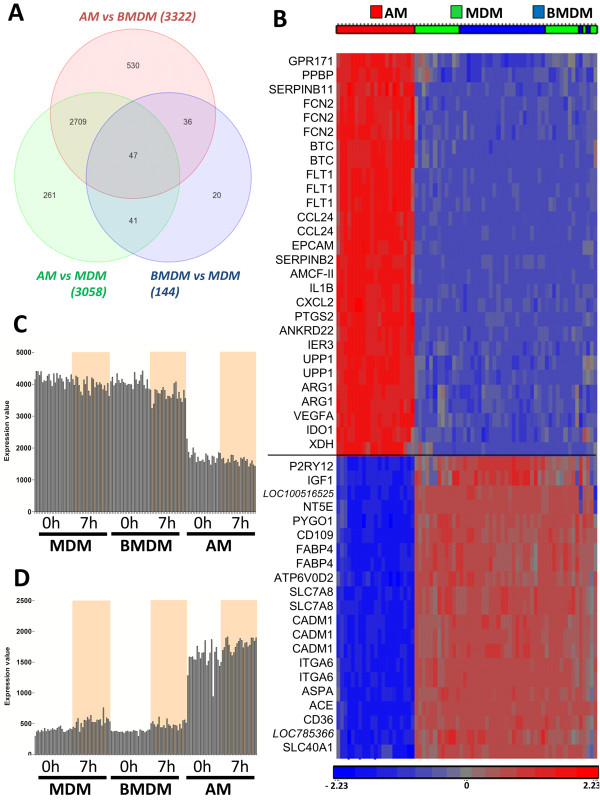
**Differences in gene expression at homeostasis between the compartments.** Microarrays were analysed using the Partek software. **(A)** Genes DR with a p adj. value < 0.01 and fold change > 2 or <-2 between the 3 types of macrophages at homeostasis (0h) were combined into a VENN graph. AM and MDM have 3,058 DR genes (green circle), AM and BMDM have 3,322 DR genes (red circle). There are 2,709 genes in common between the two lists. BMDM and MDM (blue circle) have only 144 genes DR, highlighting the closeness between these two types of derived-macrophages. **(B)** We ran a 2-way ANOVA test including the 2 variables: time-point and cell compartment). Genes that are the most differentially expressed between AM and MDM/BMDM (red, green and blue respectively) at 0 h were selected (p adj. value < 0.01 and fold change > 30 or <-30). The blue colour in the heat-map represents down-regulation of the gene and the red up-regulation of the gene in function to the average expression of the probeset. AM were found to express more VEGFA and its receptor FLT1, IDO1 and IL1B. BMDM and MDM expressed more genes related to adherence like integrin alpha 6 (ITGA6) and cell adhesion molecule 1 (CADM1). This result concurs with the different clusters found with Biolayout. Cluster 14 **(C)** includes genes highly expressed in MDM and BMDM such as ACE, where cluster 02 **(D)** is made of genes mostly expressed in AM.

To confirm the apparent difference between the macrophage populations, we analysed the data using an ANOVA method. 3,322 probesets (fold change >2 or < -2; p adjusted value < 0.01) distinguished AM from BMDM and 3,058 distinguished AM from MDM. Only 144 probesets (fold change >2 or < -2; p adjusted value < 0.01) distinguished the cultivated macrophages; 69 elevated in MDM and 75 in BMDM, essentially confirming the similarity of the populations indicated by PCA analysis. The genes that distinguished BMDM from MDM were expressed at low levels and are probably due to minor contamination with other cells: lymphocytes within the MDM and fibroblasts within the BMDM. The list included genes such as T-cell receptor gamma chain, alpha chain for the MDM and collagen type I and collagen type IV for the BMDM (list in Additional file 2). As shown in Figure 4A, most probesets differentially expressed between AM-MDM and AM-BMDM are shared (n=2,709) and only 47 probesets are differentially expressed in all 3 types of macrophages. Therefore, we selected the genes (n=49, p<0.01) that were at least 30-fold differentially-expressed between AM and BMDM and created a heat-map in Figure 4B. Most of the BMDM/MDM-specific genes are part of cluster 14 (Figure 4C), and include genes such as cell-adhesion molecule 1 (CADM1), integrin alpha 6 (ITGA6), CD36 and the insulin-like growth factor 1 (IGF1). IGF1 is known to be CSF-1-inducible [18]. The genes that are restricted to AM are part of cluster 02. The genes expressed specifically in AM (Figure 4D) included the alveolar macrophage-derived chemotactic factor-II (AMCF-II), the chemokine (C-C motif) ligand (CCL) 24, indoleamine 2,3-dioxygenase (IDO) 1, IL1 beta, the vascular endothelial growth factor (VEGF) A and its receptor (FLT1).

### Differential regulation of LPS-responsive genes in AM

**Figure 5:**
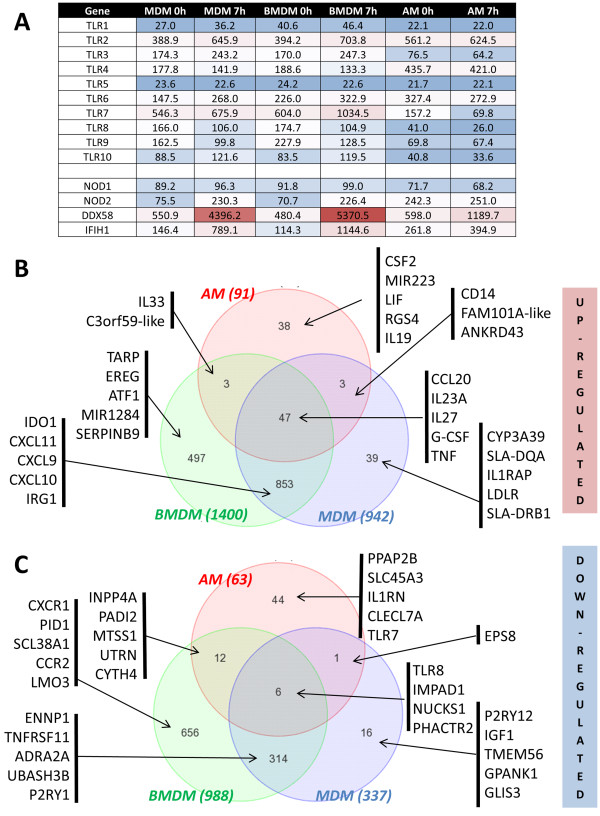
**Gene expression of different macrophages after LPS stimulation. (A)** Gene list of known PRRs in the 3 compartments at 0 and 7 h, including 10 TLR and the 4 intracellular receptors NOD1, NOD2, DDX58 (also known as RIG-1) and IFIH1 (also known as MDA5). Genes are highlighted in a scale of colour: blue for low expression (< 150), white for intermediate and red when highly expressed (> 500). Values are the mean (+/- SED) of a minimum of 23 pigs and of all probesets associated with this gene. **(B-C)** Genes differentially expressed with a p adj. value < 0.01 and fold change > 2 **(B)** or <-2 **(C**) in the 3 types of macrophages between 0h and 7h after LPS stimulation. We selected probesets DR within each compartment: AM (red), BMDM (green) and MDM (blue) with p adjusted value < 0.01 and fold change > 2 or < -2 and combined into a VENN graph. AM have 91 gene significantly up-regulated after LPS stimulation, 1,400 for BMDM and 942 for MDM. AM have 63 gene significantly down-regulated, 988 for BMDM and 337 for MDM. In order to illustrate the VENN graph, 5 of the top genes of each group are included. The total list of genes of each group is presented in Additional file 3.

PAMPs are recognised by several classes of receptors, including the toll-like receptors (TLR) and intra-cytoplasmic receptor such as nucleotide-binding oligomerization domain-containing protein (NODs) or retinoic acid-inducible gene 1 (RIG-I, also known as DDX58). As the TLRs and NODs are highly polymorphic in pigs at the protein level [19-21], we considered the possibility that they might also be differentially expressed between breeds. However, there was no evidence for differential gene expression amongst the 25 animals surveyed. There was evidence of selective expression in different macrophage populations (Figure 5A). TLR3, 7, 8 and 9 were more highly-expressed in MDM and BMDM. In contrast, TLR4 and TLR2 were highly expressed in AM. TLR6 and TLR1 proteins can both heterodimerize with TLR2 [22] but have different expression. TLR1 was barely detected, but TLR6 was selectively expressed in AM.

We selected the genes significantly regulated by LPS in AM, BMDM and MDM with a p adj. value <0.01 and a fold change >2 or <-2 (Figure 5 B, C respectively). There was again a substantial overlap between these lists in BMDM and MDM. These gene lists include the up-regulation of IDO1, IRG1 CXCL11, CXCL9 and CXCL20. As expected the 3 types of macrophages share significant up-regulation of genes encoding inflammatory mediators such as TNF, CCL20, IL23A, IL27 and G-CSF. A small set of genes induced only in the AM included GM-CSF (CSF2), LIF or IL19. The former is of interest because of the extensive literature on the specific function of GM-CSF in lung macrophage homeostasis [23]. IL19 has also been implicated in lung injury in septic shock [24]. As already shown in Figure 3, AM have a higher basal expression of inducible genes such TNF suggesting that they are primed for an inflammatory response. Indeed, there were only 91 genes significantly up-regulated in AM after LPS stimulation compare to the 1,400 in BMDM and 942 in MDM. The complete list of DR genes in the 3 populations of macrophage after LPS stimulation is listed in the Additional file 3.

### Breed-specific variation in macrophage gene expression

**Figure 6:**
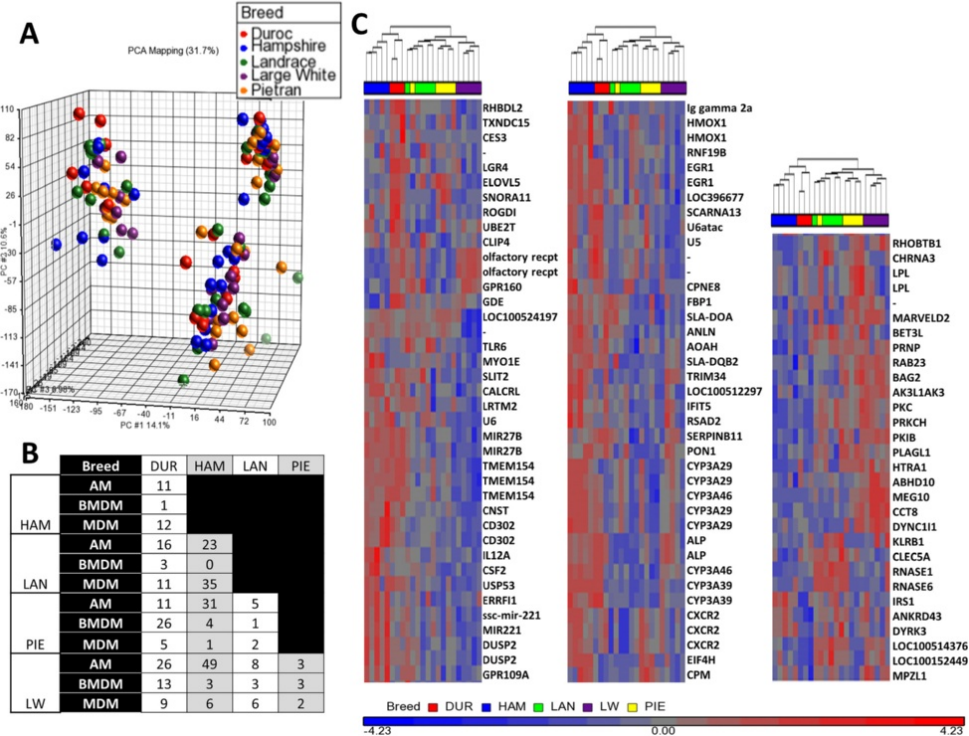
**Genes differentially regulated between the 5 breeds. (A)** 140 *Snowball* Affymetrix microarrays were used to create a principal components analysis (PCA) in function of breeds (DU in red, HAM in blue, LR in green, LW in purple and PIE in yellow). Although the PCA showed again clusters in function of the compartment and the timing, no cluster specific to breed could be observed **(B)** 3-way ANOVA test showed a small number of genes DR in function of the breed. The table includes the number of genes DR between breeds (p < 0.01 , FC >3 or <-3) after 7 h of LPS stimulation. **(C)** All the genes differentially expressed in AM were grouped into a heat-map. Duplicated probesets were removed, a total of 108 different genes were plotted (p <0.01 and fold change >3 or <-3). The blue color in the heat-map represents down-regulation of the gene and the red up-regulation of the gene in function to the average expression of the probeset.

One of our goals was to identify the set of macrophage-expressed genes that have diverged under natural and artificial selection in the pig and/or to identify individual variation that might be exploited in breeding for increased disease resistance or tolerance. In addition to Figure 1L where we showed no significant difference in TNF production between the 5 breeds for BMDM and MDM, we used a PCA on the totality of the microarrays and found no obvious clustering depending on the breeds (Figure 6A). In order to identify any breed-dependent difference in the gene expression during inflammation, we analysed all the samples (AM, BMDM and MDM) after 7 hours of LPS stimulation. We applied the stringent test of a p adjusted value <0.01 and a fold change (FC) >3 or <-3 (Figure 6B). The spectrum of differential responses amongst breeds for alveolar macrophages is shown in (Figure 6C). Only a small number of innate immune-related cytokine/chemokines genes were found differentially expressed between the breeds, including IL12A and CSF2 (GM-CSF) which were more abundantly expressed in HAM than in LW and PIE. Some genes encoding innate immune receptors are also DR amongst the breeds. TLR6 had a higher expression level in LR compared to LW and the C-type lectin CD302 expression was higher in HAM than in PIE. In addition, the IL-8 receptor beta, CXCR2, was weakly expressed in the LR breed compare to 3 other breeds (DU, HAM and PIE). Other interesting genes are DR between the breeds. The eukaryotic translation initiation factor 4H (EIF4H) was highly expressed in the Hampshire and Piétrain breed. Swine Leukocyte Antigen (SLA) (i.e. Major Histocompatibility Complex) genes (DOA, DQB2) were more highly expressed in Duroc and Hampshire in comparison to Landrace, Large White and Piétrain. These 3 breeds show a small number of DR genes, as shown in Figure 6B. In all of the comparisons, HAM and DU clustered separately from LR, LW and PIE, consistent with evidence from genomic comparisons [14]. The full list of genes DR between breeds in AM, BMDM and MDM is presented in Additional file 3. The heat-map of probesets DR between breed in MDM and BMDM are presented in Additional file 4.

### Comparison with other species: the pig as a convenient model for human disease

**Figure 7:**
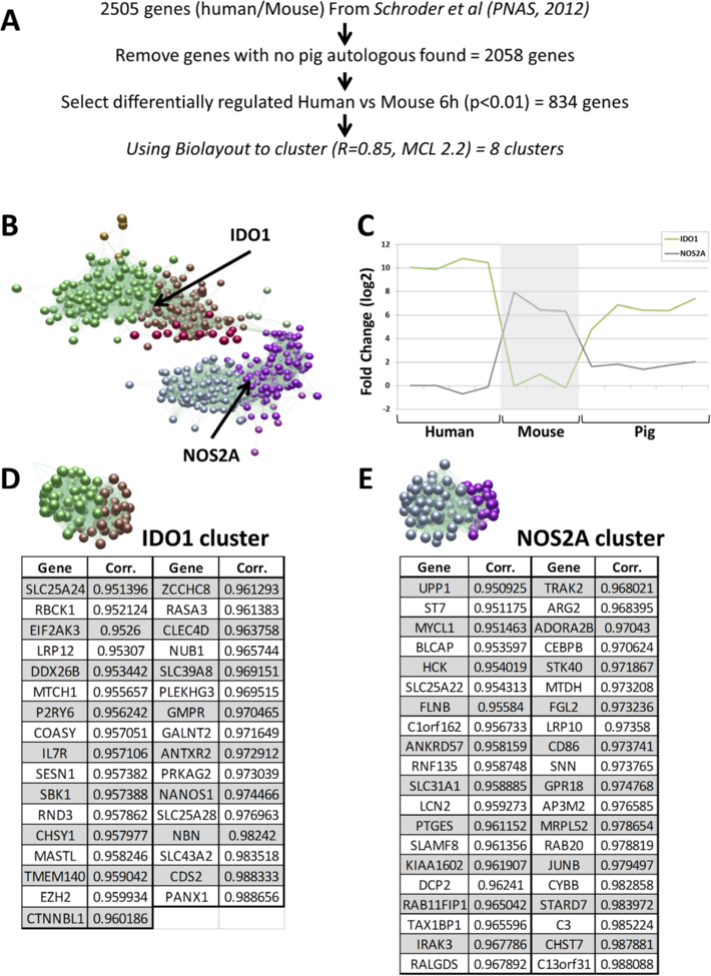
**+ Inter-species Comparison. (A)** Using the human-mouse comparison data from Schroder et al.[5], we kept 2,058 genes with a known pig orthologue. From this list, we selected 834 genes for the analysis that are significantly different between human and mouse after 6h of LPS stimulation (p<0.01). **(B)** Using Biolayout 3D, we could cluster genes expression into 8 groups using a Pearson correlation (R=0.85, MCL=2.2). **(C)** Expression of the porcine IDO1 and NOS2A genes are similar to their expression in humans, in comparison to mouse. The fold change between 0 h – /7 h LPS (in log2) of human (n=4), mouse (n=3) and pig macrophages (5 breeds, 5 animals per breed) are shown. **(D)** All neighbouring nodes of IDO1 were selected (142 genes) and 33 genes were found having a correlation higher than 0.95 with IDO1. **(E)** We selected the 143 neighbours of NOS2, genes sharing the same expression profile. From this list, 40 genes had a correlation with NOS2A > 0.95.

To compare pig macrophages with mice and human, we selected 2,505 LPS-regulated genes identified in a previous study [5]. After removing 447 genes for which no porcine orthologous genes have been annotated on the Snowball microarray, we selected 834 genes that were DR between mouse and human after 6 hours stimulation of LPS, with a p < 0.01, in the earlier study (Figure 7A). We then clustered these genes using Biolayout (R=0.85, MCL 2.2 (Figure 7B). These clusters segregate into 2 superclusters. Cluster 1 contains IDO1 (green line) which is highly up-regulated in human and pig macrophages in response to LPS, whereas Cluster 4 contains NOS2A (grey line) which is LPS-inducible only in mouse macrophages. Cluster 1 include genes such as DDX58 (also known as RIG-I). Cluster 4 (168 nodes) includes various genes playing a role in inflammation such as IRAK3, IL12B, IL18, IL6 (Figure 7C). Amongst the 142 genes sharing the profile of IDO1, 33 had a correlation >0.95 (Figure 7D). This set included GMPR, IL7R, SLC25A28, identified previously using a more limited platform and smaller dataset [16]. The mouse-specific pattern exhibited by NOS2A was shared by 143 other genes, 40 of which, including ARG2 or CD86, had a correlation > 0.95 (Figure 7E). The full dataset of correlated expression across species is provided in Additional file 5.

### Investigation on the inter-individual differences between pigs

**Figure 8:**
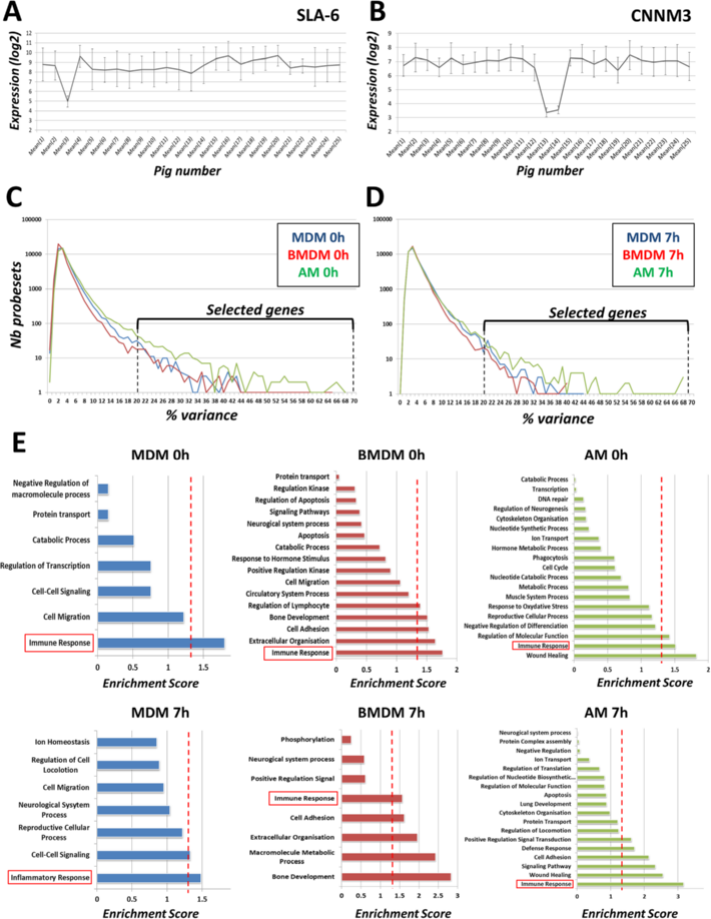
**Inter-individual differences between pigs.** The mean expression of the probesets in AM, BMDM and MDM combined, +/- LPS, of the 25 pigs were analysed. Two striking examples are the SLA-6 **(A)** and CNNM3 **(B)** genes. In one Landrace pig (pig number 3) the expression value of SLA-6 is significantly lower than the others. For the gene CNNM3, two Piétrain pigs (pigs 13 and 14) have a lower expression compared to the others pigs. These differences suggest a natural null-mutation of the genes. Number of probesets in function of their variance are graph for cells at 0 h **(C)** and 7 h **(D)** For each type of cell (MDM in blue, BMDM in red and AM in green), the average expression of each probesets has been calculated with its SED. Percentage of variance (SED/Average*100), rounded-up to a whole number, was plotted. Most of the probesets have a % of variance <10. We were interested in the list of probesets having a high variability between pigs (>20%). **(E)** We analysed this list of probesets for each type of cell and timing with the DAVID algorithm (http://david.abcc.ncifcrf.gov) in order to cluster them in function of their biological process (lowest clustering option), using the list of expressed gene as background. The red dashed line represents the usual enrichment score threshold (1.3). A score > 1.3 is definitively playing an important role. In each case, the cluster of “immune response” was over this threshold.

We examined the array data to identify candidate null mutations. In keeping with previous evidence of variable expression [25], we found that one pig had no expression of swine leukocyte antigen 6 (SLA6), the major histocompatibility complex in pigs (Figure 8A) and two Piétrain pigs had substantially lower expression of the cyclin M3 (CCNM3) gene than the rest of the animals (Figure 8B). To identify genes with highly variable expression between individuals, we calculated the average expression and the SED of all probesets for all 6 conditions (3 types of cells and 2 stimulation timepoints). Then, we analysed the percentage of variance (SED/Mean*100) of each probeset. The full list of probesets with their variance is provided in Additional file 6. The majority of genes have a percentage variance < 10.

The variance data are plotted in Figure 8C and D. The genes with highest percentage variance (between 25–50%) are clearly enriched for immune function. In BMDM and MDM the most variable gene is CXCL10 followed by interferon beta, interferon-induced protein with tetratricopeptide repeats (IFIT) 1, DDX58, IDO1, CXCL11, IL7R and IL1RN. In AM, the top genes (>50%) are linked to the immunoglobulin chain, due to the small contamination of B cell from the alveolar lavage. Outside of this set, genes with a variance between 20–50% included IL33, CCR2, IL23A, IGF1, CXCL9 and CXCL10. Genes with a variance > 20% were analysed using the DAVID functional annotation webtool [26] [http://david.abcc.ncifcrf.gov]. For each compartment and time-point (a total of 6), the biological process clusters were given an enrichment score, ranking the overall importance of the annotation term group (Figure 8E). The score of 1.3 is taken as the threshold for functional importance (red line). Clearly, immune response genes are identified in every comparison.

## Discussion

In this study, we developed ways to harvest macrophages from the lungs, blood and bone-marrow of pigs and to freeze them for later use. This approach provides a convenient basis for analysis of the genetic variation in host pathogen interaction using *in vitro* challenge models. Morphology, viability, TNF production and expression of surface markers (CD14 and CD16) were unaltered by cryopreservation. Our analysis allowed us to compare 5 different breeds and 3 different compartments (bone-marrow, blood and lungs) in terms of their gene expression profiles and responses to LPS.

The basal gene expression pattern in alveolar macrophages (AM) was clearly distinct from the patterns in monocyte-derived or bone marrow-derived macrophages, regardless of breed. The differences include a relatively high basal expression of IDO1, CXCL2, CCL24 and IL1B. Interestingly, we also found that the genes encoding VEGFA and its receptor FLT1 were also highly expressed in AM. Alveolar macrophages do express the receptor for the macrophage growth factor, CSF-1 (CSF1R). However, unlike most tissue macrophages, in the mouse they do not depend upon continued CSF1R signalling [27]. In the op/op CSF-1 deficient mouse, lung macrophage numbers correct with age [28] and Flt1 signalling has been attributed a role in age-dependent correction of the bone phenotype in op/op mice [29]. We suggest that VEGF might have specific roles in alveolar macrophage homeostasis.

Pattern recognition receptors also distinguished the macrophage populations regardless of breed background. The high levels of lectin-like receptors noted previously [15] could contribute to elimination of particulate material in the airways, including bacteria and fungi. AM expressed more TLR4 (Figure 5), suggesting that AM would target mostly bacteria detection. In contrast to AM, the BMDM and MDM expressed more TLR3, TLR7, TLR8 and TLR9 as well as RNA intracellular receptors DDX58 (RIG-1) and IFIH1 (MDA5) suggesting a co-regulated cluster of genes involved in virus detection. There is, of course, a fundamental difference between the AM and the two populations derived by cultivation in CSF-1, the MDM and BMDM. The latter cells expressed cell-cycle-related genes and may also be cell cultured adapted. For the purpose of genetic studies, the culture systems have the advantage that they largely eliminate the effect of in vivo environment including health status, and this is reflected in the relatively consistent gene expression profiles. Nevertheless, Fejer et al. [30] have recently emphasised the fact that the phenotype of alveolar macrophages in mice can be replicated in vitro to some extent by cultivation of bone marrow cells in GM-CSF, as opposed to CSF-1.

We also compared the inflammatory response in 5 different breeds and identified a small set of genes that could contribute to different disease resistance between breeds. Landrace pigs expressed substantially less IL-8R beta (CXCR2) than the other breeds (Duroc, Hampshire and Piétrain). Ait-Ali et al. [11] reported that Landrace alveolar macrophages were more resistant to PRRS virus replication and released large amounts of TNF and IL8 into the supernatant. It remains to be seen whether differential expression of the IL8 receptor contributes to this biology. The number of genes differentially expressed between the breeds was relatively small (Figure 6). There was much greater variation between individuals within breeds, which also urges caution upon studies of breed differences based upon relatively small group sizes. Amongst the differences was the apparent absence of expression of SLA6 (Figure 8) and highly variable expression of SLA-DOA. These differences might be associated with polymorphic variation in miRNA recognition sites reported elsewhere [31]. Significant levels of protein-coding polymorphism have already been reported amongst pig pattern intracellular and extracellular recognition receptors [19,32]. It is striking that such genes are DR between the macrophage populations, and highly variable between individuals. It remains to be determined whether such variation is heritable and can be linked to SNP markers to allow selective breeding. The method we have applied herein, which can be performed on blood and does not require pathogen challenge of the animal, could potentially permit *in vitro* screening of breeding animals for optimal innate immune responsiveness.

In keeping with our earlier findings, now applied to a much larger data set and macrophages from multiple sites including monocyte-derived macrophages, pigs and humans share innate immune responses that are absent from rodents, and conversely, mice induce pathways that are not shared with large animals [5,16]. The index genes for these classes of genes are IDO1, expressed only in human and pigs, and NOS2A, expressed only in mouse. Using Biolayout *Express*^3D^, we found clusters of genes that share the same expression patterns with these index genes (Figure 3). Analysis of the draft pig genome has highlighted numerous candidate genes underlying human pathology [14]. The findings herein emphasise the applications of understanding pig innate immunity for biomedical research as well as improved livestock production and animal health [10].

## Conclusions

We have examined the differences in cellular markers and gene expression between multiple macrophage populations from 25 pigs of five breeds. The results indicate that individual pigs vary most markedly in their expression of immune-associated inducible genes, whereas there are no major breed-dependent variations. The findings, using the LPS stimulation as an inflammation trigger, suggest that there has been relatively little selection of pigs breeds for immune-associated traits. We show also that pig macrophages are human-like in their inducible gene expression profile, and the pig may therefore provide a superior model for dissection of human inflammatory diseases.

## Methods

### Animals

5 pigs at 8–12 weeks old (3 females, 2 males) of 5 different breeds were used in this study: the genome reference breed Duroc (DU), Piétrain (PIE), Large White (LW), Landrace (LR) and Hampshire (HAM). All the pigs spent at least 2 weeks in the same facility at rest before experimentation. Animals have not shown any signs of any infections, did not received any vaccinations and none of the female pig were pregnant. All animal studies were conducted according to University of Edinburgh Guidelines and were approved by the Institutional Ethics Committee.

### Cell isolation and cryopreservation

Pigs were injected with a mixture of ketamine (6 mg/kg) and azaperone (1 mg/kg), left undisturbed for 10–15 min then killed by captive bolt. Approximately 400 ml of blood was drawn by cardiac puncture, using a blood bag (Sarstedt, Nümbrecht, Germany). Lungs were then removed and kept on ice after clamping the trachea to avoid blood contamination. Finally, 5 posterior ribs from each side of the animal were removed and kept on ice. To isolate mononuclear cells (PBMC), the blood was centrifuged at 1200 g for 15 min with no brake and the buffy coat was removed and mixed with an equal volume of RPMI-1640 medium (Sigma-Aldrich, USA). PBMC were separated further using Lymphoprep (Axis-Shield, Norway) and centrifuged at 1200 g for 25 min with no brake. The mononuclear cell fraction was washed twice with phosphate buffered saline (PBS) (centrifuged 5 min at 600 g then 400 g). Bone marrow cells were harvested as previously described [16]. In short, the ribs were cleaned with 70% (v/v) ethanol and both extremities were cut. The bone was flushed with RPMI-1640 (containing 5 mM EDTA to prevent clotting) using a 20 ml syringe and a bone marrow biopsy/aspirate needle (Cardinal Health, USA). Alveolar macrophages were extracted by flushing the lungs twice with 500 ml of PBS). The volume recovered was filtered (100 μm) and centrifuged (10 min, 400 g). Red cells were removed by suspension in 5 ml of lysis buffer (10 mM KHCO_3_, 155 mM NH_4_Cl, 0.1 M EDTA, sterile 0.2 μM filtered) for 5 min followed by PBS wash. All three type of cells were finally centrifuged at 400 g for 5 min and the pellet was collected, re-suspended in freezing medium (90% FCS, 10% DMSO) and was slow frozen at -80°C in an isopropanol bath. Cells were retained at -155°C for long term storage.

### Cell culture

Cells were recovered from -155°C by quickly thawing them at 37°C, then slowly diluting the freezing medium by dropwise addition of 40 ml of warm PBS over 2–3 min to avoid the shock of sudden dilution of DMSO. In order to obtain macrophages, bone-marrow cells and PBMC were cultured 5–7 days in large 100 mm^2^ sterile petri dishes in 20 ml of complete medium: RPMI-1640, Glutamax supplement (35050–61; Invitrogen), 10% heat-inactivated FCS (PAA Laboratories), penicillin/streptomycin (15140; Invitrogen, Paisley, UK) in the presence of rhCSF-1 (1×10^4^ units/ml; a gift of Chiron, Emeryville, CA). Bone marrow-derived macrophages (BMDM), monocyte-derived macrophages (MDM) or alveolar macrophages (AM) were then seeded at 1×10^6^ cells/ml in 6-wells plates in complete medium with rhCSF-1 and left overnight. The next day, non-adherent cells were removed, fresh complete medium was added and cells were stimulated with LPS from *Salmonella enterica* serotype minnesota Re 595 (100 ng/ml; L9764; Sigma-Aldrich).

### RNA extraction

RNA was extracted from AM, BMDM and MDM at 0 h and 7 h after LPS stimulation, using Amsbio RNA-Bee kit, as specified by the manufacturer (Amsbio, Abingdon, U.K.). RNA concentration was measured using ND-1000 Nanodrop (Thermo Scientific). The quality was controlled by running the samples on the RNA 6000 LabChip kit (Agilent Technologies, Waldbronn, Germany) with the Agilent2100 Bioanalyzer in which samples are assigned an integrity classification from 10 (intact RNA) to 1 (highly degraded) by the 2100 Bioanalyzer expert software.

### Snowball porcine micro-array

Total RNA was prepared for hybridization using the Ambion's WT Expression Kit (Affymetrix, Santa Clara, CA), following the manufacturer’s instructions, except for the input amount of RNA (500 ng input instead of the recommended 100 ng). We then hybridized in a random order to the Affymetrix Snowball Porcine Array [15] by ARK-Genomics [http://www.ark-genomics.org]. This array was designed by us, and each probeset is composed of an average of 11 probes dispersed along the transcript to avoid any impact of polymorphism on detection. Statistical analysis of the array data utilised Partek Genomic Suite (Partek, St. Louis, USA). For network analysis, the normalised array data were uploaded to the software Biolayout *Express*^3D^ [http://www.biolayout.org/] as described previously [15,33]. The data from the microarray are available at [http://www.macrophages.com] and at Gene Expression Omnibus NCBI [http://www.ncbi.nlm.nih.gov/geo/] - serie GSE45145.

### ELISA

Supernatants from stimulated cells were harvested and stored frozen at -25°C until assayed in a single batch. Porcine TNF was measured by ELISA, following the manufacturer’s instruction (Duoset DY690B; R&D Systems, Minneapolis, MN).

### Flow cytometry

Cells were incubated 15 min in high-block solution (PBS, 0.1% sodium azide, 2% FCS, 0.1% BSA) then washed with low-block solution (PBS, 0.1% sodium azide, 0.2% FCS, 0.1% BSA). Macrophages were stained with either a mouse anti-pig CD14 (clone MCA1218, 1:50; AbD Serotec), a mouse anti-pig CD16 (clone MCA1971, 1:200; AbD Serotec), or an IgG2b or IgG1 isotype control (MCA691 and MCA928PE; AbD Serotec; same concentration as primary Ab) in Low Block. The cells were then washed and resuspended in 500 μl Low Block. Data (10K cells) were acquired using a CyAn ADP Analyzer (Beckman Coulter, High Wycombe, U.K.) and analyzed with Summit software (v4.3).

## Competing interests

The authors declare they have no competing interests.

## Authors’ contributions

RK performed most of the cell isolation and analysis, with assistance from LF. AD carried out the array profiling and assisted with informatic analysis. DB and CKT contributed to the annotation of the pig array. TF contributed to informatics analysis. DPS, ALA and DAH designed the project; RK and DAH wrote the paper. All authors read and approved the final manuscript.

## Supplementary Material

Additional file 1 **Full list of clusters of pig macrophage gene expression using Biolayout.** We analysed the 140 pigs micro-arrays (BMDM, MDM and AM - at 2 timepoint 0 h and 7 h using a Pearson correlation (R=0.91). The graph was composed of 5203 nodes and 29799 edges. In order to more easily differentiate the data from background noise, probesets with expression <50 in all samples have been removed from the analysis. List of all 505 clusters, using a MCL algorithm (MCL = 2.2), with the probeset number, the gene name, the gene description and the cluster number. Click here for file

Additional file 2 **Full list of gene differentially regulated between compartment at homeostasis.** Microarrays were analysed using the Partek software. (1) 49 probesets DR with a p adj. value < 0.01 and fold change > 30 or <-30 between AM and BMDM at homeostasis (0h) were listed. (2) List of the 144 probesets DR between MDM and BMDM at 0 h (p adj. value < 0.01 and fold change > 2 or <-2). (3) List of the 3322 probesets DR between AM and BMDM at 0h (p adj. value < 0.01 and fold change > 2 or <-2). (4) List of the 3058 probesets DR between AM and MDM at 0h (p adj. value < 0.01 and fold change > 2 or <-2). Click here for file

Additional file 3 **DR genes after LPS stimulation.** Genes differentially expressed with p adj. value < 0.01 and upregulated (1) or down-regulated (2) (fold change > 2 or < -2) in the 3 types of macrophages between 0 h and 7 h after lps stimulation. Genes DR between the 5 breeds (p <0.01 and fold change >3 or <-3) in AM (3), BMDM (4) and MDM (5). Click here for file

Additional file 4 **Genes differentially regulated between the 5 breeds in BMDM and MDM.** As in Figure 6, list of genes DR in BMDM and MDM after 7 h of LPS stimulation were grouped and included into a heat-maps (A and B respectively). DU is in red, HAM in blue, LR in green, LW in purple and PIE in yellow. The blue colour in the heat-map represents down-regulation of the gene and the red up-regulation of the gene in function to the average expression of the probeset. Duplicated probesets were removed, a total of 38 different genes for BMDM and 65 for MDM were plotted (p <0.01 and fold change >3 or <-3). Click here for file

Additional file 5 **Inter-species Comparison.** (1) Using the human-mouse comparison data from Schroder et al. [5], we kept 2,058 genes with a known pig orthologue. From this list, we selected 834 genes for the analysis that are significantly different between human and mouse after 6 h of LPS stimulation (p<0.01). (2) Using Biolayout 3D, we could cluster genes expression into 8 groups using a Pearson correlation (R=0.85, MCL=2.2). (3) List of all neighbouring nodes of IDO1 (142 genes) and NOS2 (143 genes). 33 genes were found having a correlation higher than 0.95 with IDO1 and 40 genes had a correlation with NOS2A > 0.95. Click here for file

Additional file 6 **The table have been sorted for each type of cell (MDM in blue, BMDM in red and AM in green) from the probeset with the largest variance to the smallest.** The % of Variance id in the SED/Mean*100. Click here for file
