# Supplementary figures and images for: The impact of breed and tissue compartment on the response of pig macrophages to lipopolysaccharide
Source: BMC Genomics. 2013 Aug 28;14:581. doi: 10.1186/1471-2164-14-581 (PMC3766131; doi:10.1186/1471-2164-14-581)

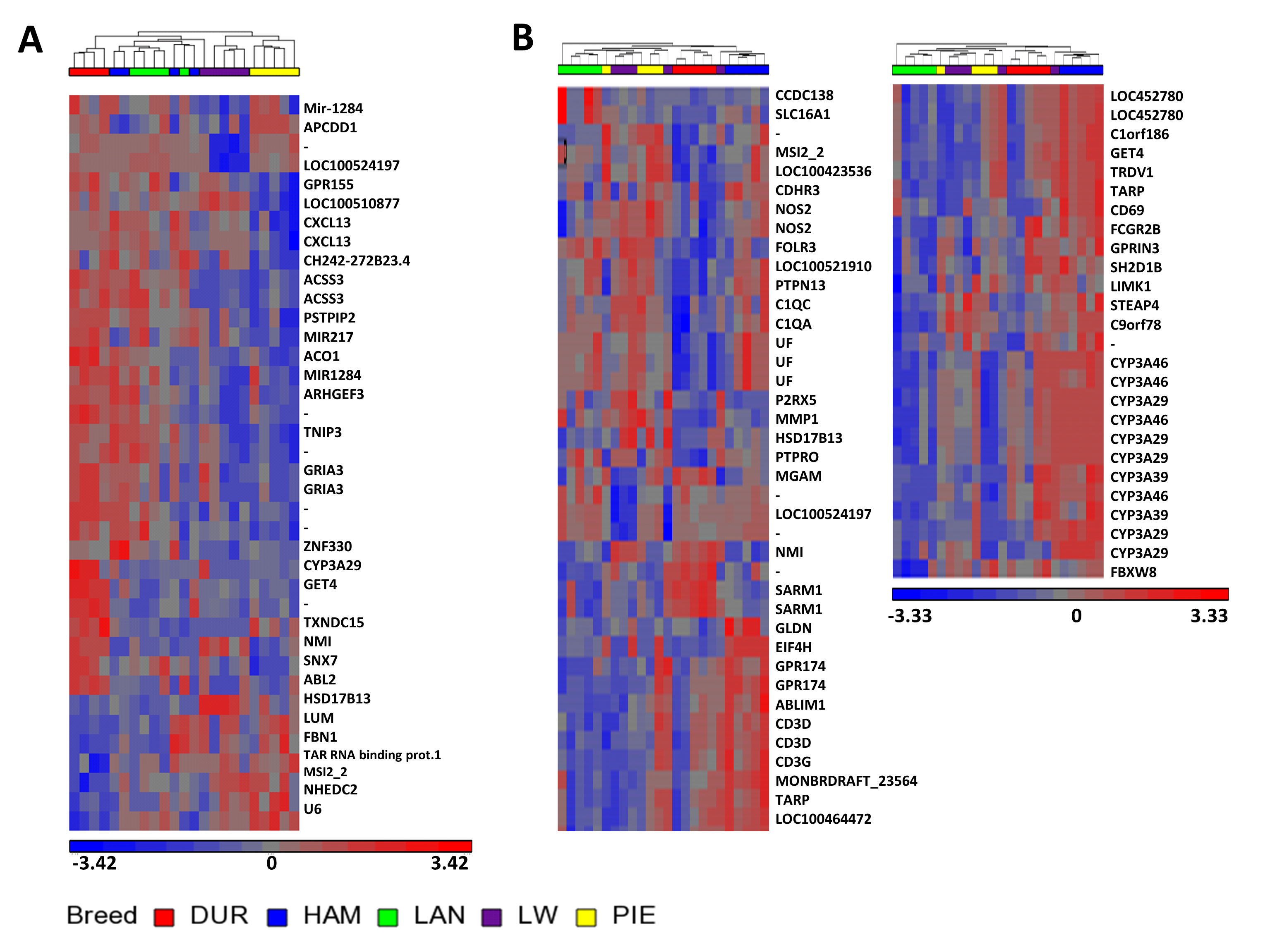

Supplement: Additional file 4 — Genes differentially regulated between the 5 breeds in BMDM and MDM. As in Figure 6, list of genes DR in BMDM and MDM after 7 h of LPS stimulation were grouped and included into a heat-maps (A and B respectively). DU is in red, HAM in blue, LR in green, LW in purple and PIE in yellow. The blue colour in the heat-map represents down-regulation of the gene and the red up-regulation of the gene in function to the average expression of the probeset. Duplicated probesets were removed, a total of 38 different genes for BMDM and 65 for MDM were plotted (p <0.01 and fold change >3 or <-3). [file 1471-2164-14-581-S4.tiff]
